# Characterization of Inclination Analysis for Predicting Onset of Heart Failure from Primary Care Electronic Medical Records

**DOI:** 10.3390/s23094228

**Published:** 2023-04-24

**Authors:** Federica Guida, Marta Lenatti, Karim Keshavjee, Alireza Khatami, Aziz Guergachi, Alessia Paglialonga

**Affiliations:** 1 Dipartimento di Elettronica, Informazione e Bioingegneria (DEIB), Politecnico di Milano, 20133 Milan, Italy; 2 Cnr-Istituto di Elettronica e di Ingegneria dell’Informazione e delle Telecomunicazioni (CNR-IEIIT), 20133 Milan, Italy; 3 Institute of Health Policy, Management and Evaluation, University of Toronto, Toronto, ON M5T 3M6, Canada; 4 Ted Rogers School of Management, Toronto Metropolitan University, Toronto, ON M5G 2C3, Canada; 5 Ted Rogers School of Information Technology Management, Toronto Metropolitan University, Toronto, ON M5G 2C3, Canada; 6 Department of Mathematics and Statistics, York University, Toronto, ON M3J 1P3, Canada

**Keywords:** disease prediction, electronic medical records (EMR), heart failure, inclination analysis, longitudinal data, machine learning

## Abstract

The aim of this study is to characterize the performance of an inclination analysis for predicting the onset of heart failure (HF) from routinely collected clinical biomarkers extracted from primary care electronic medical records. A balanced dataset of 698 patients (with/without HF), including a minimum of five longitudinal measures of nine biomarkers (body mass index, diastolic and systolic blood pressure, fasting glucose, glycated hemoglobin, low-density and high-density lipoproteins, total cholesterol, and triglycerides) is used. The proposed algorithm achieves an accuracy of 0.89 (sensitivity of 0.89, specificity of 0.90) to predict the inclination of biomarkers (i.e., their trend towards a ‘survival’ or ‘collapse’ as defined by an inclination analysis) on a labeled, balanced dataset of 40 patients. Decision trees trained on the predicted inclination of biomarkers have significantly higher recall (0.69 vs. 0.53) and significantly higher negative predictive value (0.60 vs. 0.55) than those trained on the average values computed from the measures of biomarkers available before the onset of the disease, suggesting that an inclination analysis can help identify the onset of HF in the primary care patient population from routinely available clinical data. This exploratory study provides the basis for further investigations of inclination analyses to identify at-risk patients and generate preventive measures (i.e., personalized recommendations to reverse the trend of biomarkers towards collapse).

## 1. Introduction

Heart Failure (HF) is a progressive chronic disease with substantial associated burden worldwide, particularly in the elderly [1]. The incidence of HF is projected to increase in the future due to population aging and to growing incidence of diabetes, obesity and hypertension, that are well-known comorbidities of HF [1,2]. Early detection of HF is important as this pathology is associated with a high level of disability, health care costs, and mortality [3]. However, an early detection of HF may be difficult because the early symptoms of HF are often overlooked or attributed to other diseases and conditions. For example, dyspnea is a common symptom of HF that can lead to a diagnosis of chronic obstructive pulmonary disease, a common comorbidity of HF [4]. 

Machine learning algorithms, such as tree-based models, logistic regression, support vector machines, and deep learning approaches such as convolutional neural networks and recurrent neural networks, have been applied for predicting HF [5,6,7,8,9,10,11]. Overall, these studies showed promising results, with accuracy ranging from about 65% to about 83%. In general, in the abovementioned studies, the models were trained mainly on measures derived using basic aggregation functions or first-order statistics (e.g., counts, average values), and the time series of biomarkers collected from longitudinal clinical measures were not analyzed. Moreover, the majority of the machine learning models developed in the literature were trained using specific biomarkers as input features (e.g., B-type natriuretic peptide level, glomerular filtration rate, specific cardiological measures) that are not routinely collected in primary care. As such, these predictive models may lack generalizability and may not be applicable on a wide scale for HF prediction.

Considering the challenging diagnosis of HF and the usually late identification, it would be important to develop algorithms able to identify early individuals at risk of developing HF using clinical measures collected in routine real-life settings (e.g., in primary care). Due to the degenerative nature of HF, an analysis of the longitudinal measures of clinical biomarkers could potentially help identify the changes in health state earlier than other methods and in real time, for example, by integrating predictive algorithms into primary care Electronic Medical Records (EMRs) systems. 

The rationale of this study is to develop predictive models of HF that may be applied to the broad population of primary care patients and used for the early detection of the risk of developing HF. Specifically, in this study, we developed predictive models using only biomarkers that are largely available in primary care (e.g., blood pressure, cholesterol, fasting glucose) and available from primary care EMRs. Moreover, in this study, we analyzed the longitudinal measures of biomarkers in the form of time series (as opposed to common approaches using average values or aggregate measures). Specifically, we introduced an original approach based on an inclination analysis, which is an algorithm capable of predicting the long-term trends of time series based on a relatively short window of observations. In this study, the inclination analysis is used to assess the time-varying nature of the risk of developing HF and to investigate the progression of a dynamic system (i.e., the body) towards a certain state (e.g., a disease) for the ultimate aim to develop an early identification system able to detect the individual risk in real time. Such real-time applications could not only help identify patients at risk years before the onset, but could also support personalized prevention, such as through the identification of recommendations able to reverse the trend towards disease in a personalized manner, specifically by slowing down or preventing the inclination towards the disease of specific biomarkers, which are defined on an individual basis.

The inclination analysis was first designed by Kryazhimskii and Beck in 2002 [12] to model the behaviour of environmental systems (e.g., survival/collapse). The same approach was later applied to analyze financial time series [13], but to the best of our knowledge, so far, the inclination analysis has not been applied to develop health prediction models. An inclination analysis is based on a time series path-dependent stochastic process theory to predict the future behaviour of a system from past observations. At any given instant in the observation window, the state of the system is defined as either survival (i.e., improvement) or collapse (i.e., worsening), and the system is supposed to evolve towards a stable final state. The final state of the system is determined by comparing the probability of a given state (computed from the available observations) with the ‘prior’ probability of that state, computed on the basis of a hypothetical uniform distribution of observations. Such an approach might be promising in the clinical field since it is computationally simple, explainable, finely tunable to specific diseases based on the related medical guidelines, and can be used to generate preventive measures (i.e., personalized recommendations to reverse the trend of biomarkers towards collapse). However, the application of an inclination analysis to routinely collected clinical data is challenging because of the following: 

(i) Clinical data are usually not collected at a fixed sampling frequency, but they are rather collected at clinical encounters or at prescribed examinations, whereas the original formulation of the inclination analysis algorithm is based on regular time sampling. The time between two consecutive measures of a given biomarker can be of the order of hours, days, weeks, months, or even years. Therefore, to apply an inclination analysis to unevenly sampled data, we need to define measures to quantify the ‘improvement’ or ‘worsening’ of a clinical biomarker in the available (and irregular) time intervals (e.g., the difference between consecutive measures, the slope, the underlying area, or a binary variable describing the increase/decrease).

(ii) Clinical data are complex in nature as the ‘improvement’ or ‘worsening’ of a clinical biomarker is not identified by a pre-defined increase or decrease in its value, but rather, it should be identified with respect to clinically acceptable ranges. Conversely, the original formulation of the inclination analysis was defined on simpler data (i.e., environmental/financial time series) where an increase (or decrease) in value was associated with ‘improvement’ (or ‘worsening’) in a straightforward manner. Therefore, to apply an inclination analysis to clinical biomarkers, measures to identify the local state of the system in terms of ‘improvement’/‘worsening’ need to be defined. For example, information about the increase/decrease of a given observed biomarker could be combined with considerations related to clinically acceptable values.

(iii) Clinical data are multivariate in nature as multiple biomarkers are monitored in time, whereas in the original formulation of the inclination analysis, univariate time series are analysed (e.g., rodent population size in environmental behaviour prediction, exchange rates and closing stock prices in financial prediction) [12,13]. Therefore, to apply an inclination analysis to multivariate clinical data, novel approaches should be defined (e.g., classification models able to deal with multiple features per record).

In this exploratory study, we introduce for the first time an approach based on the inclination analysis to analyze multivariate biomarkers time series from routinely collected EMR data to develop predictive models of HF applicable in primary care. To address the challenges (i) and (ii), we investigate several versions of the algorithm to identify the one that predicts the final state of biomarkers time series with the highest accuracy. To address challenge (iii), we introduce a machine learning classifier to predict the onset of HF from a multivariate input based on the binary predictions (i.e., survival/collapse) of the final state of the system, as outlined in detail in the following section.

## 2. Materials and Methods

### 2.1. Dataset

The dataset used in this study includes nine biomarkers from 698 patients (349 with HF, 349 with no HF, i.e., 50% class balance), extracted from a Canadian database of primary care EMRs, namely the Canadian Primary Care Sentinel Surveillance Network (CPCSSN) [14]. The group of HF patients included 145 females (age: 73.6 ± 11.2 years) and 204 males (age: 74.2 ± 10.7 years), whereas the group of no HF patients included 183 females (age: 60.8 ± 11.7 years) and 166 males (age: 62.6 ± 12.6 years). The following biomarkers were considered: body mass index (BMI), diastolic and systolic blood pressure (dBP and sBP, respectively), fasting glucose, glycated hemoglobin (HbA1c), low-density lipoprotein (LDL), high-density lipoprotein (HDL), total cholesterol, and triglycerides.

Based on preliminary analysis, to implement inclination analysis in a reliable way, the minimum number of measures was set to five readings for each biomarker. Data were oversampled from N to 2N − 1 by halving the interval between subsequent readings and by estimating the new samples as the average value of the two adjacent readings, obtaining time series of nine or more samples. The rationale was to set a trade-off between accuracy (the longer the time series, the more accurate the estimated probability of the final state of the system from the observed data) and sampling bias (the longer the time series, the higher the chance that the patients included in the sample were older and/or were in worse health condition).

### 2.2. Inclination Analysis

#### 2.2.1. General Principles

**Figure 1:**
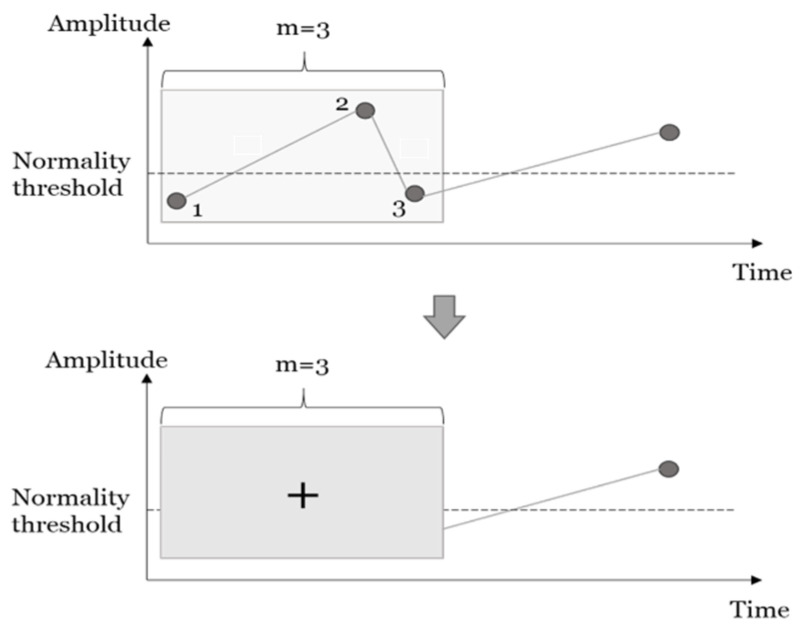
Conversion of continuous biomarker values (samples 1, 2, and 3) into binary values (local state) using a window of length *m* = 3.

In this section, a brief outline of inclination analysis is reported. A detailed description can be found in [12,13]. An exemplary representation of inclination analysis is shown in Figure 1. Given a discrete time series of biomarker measures of amplitude *x*(*k*), with *k =* 1, *…*, *N* and a time window of length *m*, the inclination analysis algorithm translated each portion of the time series (extracted using a sliding window of length *m*, including samples from *k* − *m* to *k*) into a binary value that represented the local state in *k* and was defined as +(survival) or −(collapse). In the original formulation of the inclination analysis algorithm, this binary value was defined as the sign of the differences between biomarker amplitudes in the extreme samples of the window (i.e., in reference to the example in Figure 1, the binary value was the sign of *x*_3_ − *x*_1_). In this study, several modifications to the original algorithm were introduced and tested to address clinical data characterization, as reported in Section 2.2.2 below.

The probability of a + (survival) or −(collapse) local state in the subsequent windowed portion of the time series, estimated at a discrete time instant *k + h* by shifting the window of *h* samples (with *h =* 1 in our study), was defined by introducing the critical levels *m^−^* and *m^+^* with *m^+^* + *m^−^* ≤ *m*. The critical levels *m*^−^ and *m^+^* defined the minimum number of states − and + (*n^−^* and *n^+^*, respectively) in a window of length *m* at time *k* that would lead to a stable state (i.e., to a sequence of *m* consecutive − states or a sequence of *m* consecutive + states) at time *k + h* with probability equal to 1. When *n^−^* < *m^−^* and *n^+^* < *m^+^*, the system would evolve into a− (or a+) in *k + h* with probability *r*^−^ *= R* (or *r^+^* = 1 − *R*).

The probability of each of the 2*^m^* possible final sequences of states (*P_c_*) was estimated using (1):

*P_c_* = *Z_inf_* × *q_c_*, (1)

where *q_c_* is the observed distribution of probabilities for all the possible sequences of *m* states and *Z_inf_* = *Z^l^*, where *l* is a sufficiently large number, as it will be described in Section 2.2.2. The transition probability matrix *Z* (size: 2*^m^* × 2*^m^*) described the probability of the system to evolve from any given sequence of *m* local states in the window from *k − m +* 1 to *k* to any given sequence of *m* local states in the next window (i.e., from *k − m* to *k + h*). The inclination of the system towards a given final state was estimated by comparing the probability of that final state computed from the observed distribution of states *q_c_* (e.g., *P_c_^−^* for collapse) with the ‘prior’ probability of the same state computed from a hypothetical uniform distribution of states *q* (i.e., *P^−^*). Specifically, in the original formulation of the algorithm, the predicted final state of the system was collapse if *P_c_^−^ > P^−^*, survival if *P_c_^−^ < P^−^*, and undefined if *P_c_^−^~P^−^*.

#### 2.2.2. Application of Inclination Analysis to Clinical Data

Eight versions of the inclination analysis algorithm were developed and tested in this study (Table 1).

In all clinical biomarkers considered here, with the exception of HDL, a decrease was typically associated with improved health as values below a certain threshold were desired. Specifically, for each biomarker, a normality threshold was introduced (shown in Table 2) that defined the cut-off limit of clinically desirable values (e.g., sBP < 120 mmHg, HDL > 1.5 mmol/L). For the sake of simplicity, in this section, the algorithm is described in relation to this typical behaviour of biomarkers (decrease/improvement vs. increase/worsening). By using the same principles, biomarkers with an opposite behaviour could be modeled.

To determine the local state of a given biomarker in each window of length *m* from *x*(*k − m* + 1) to *x*(*k*), a variable *Xs* was computed as shown in (2):

*Xs* = Σ*_i_* Δ*_i_*
*w_i_*, (2)

where Δ*_i_* reflects the local increase/decrease, and *w_i_* is a weighting factor computed from each pair of consecutive samples within the window *m* (i.e., from samples *x*(*k − m* + *i*) and *x*(*k − m* + *i* + 1), with *i* = 1, …, *m* – 1). The value of *Xs* computed using Equation (2) was used to determine the local state of the biomarker in the time instant *k*, specifically survival (+) if *Xs* < 0 and collapse (*−*) otherwise.

The window length *m* and the definitions of Δ*_i_* and *w_i_* used in the eight versions of the algorithm are summarized in Table 1. The eight versions of the algorithm introduced here differed based on the definitions of Δ*_i_* and *w_i_*. For example, in the original formulation of the algorithm and with reference to the example of Figure 1, *Xs* = Δ_1_ + Δ_2_, with Δ_1_ = *x*_2_ − *x*_1_ and Δ_2_ = *x*_3_ *− x*_2_, leading to a local state + (survival) in the case of biomarker decrease (i.e., improvement) within the window and to a local state *−* (collapse) otherwise. Specifically, four different definitions were tested for Δ*_i_*. The simpler definition was the same as in the original version (i.e., Δ*_i_* was defined as the difference between consecutive values) to capture information about the amount of local increase/decrease. In version #1, to capture information about the cumulative effects of a local increase/decrease, Δ*_i_* was defined as the integral-ratio, which was the ratio of the integral computed between two consecutive samples to the integral computed along the whole time series. In versions #3 and #4, to capture information about the steepness of the local increase/decrease, Δ*_i_* was defined as the slope of the segment connecting two consecutive samples, whereas in versions #5–7, Δ*_i_* was defined as the sign of the difference between two consecutive samples to capture the direction of the local increase/decrease. Accordingly, Δ*_i_* introduced a negative contribution in Equation (2) whenever the biomarker showed a local decrease and a positive contribution whenever the biomarker showed a local increase.

**Figure 2:**
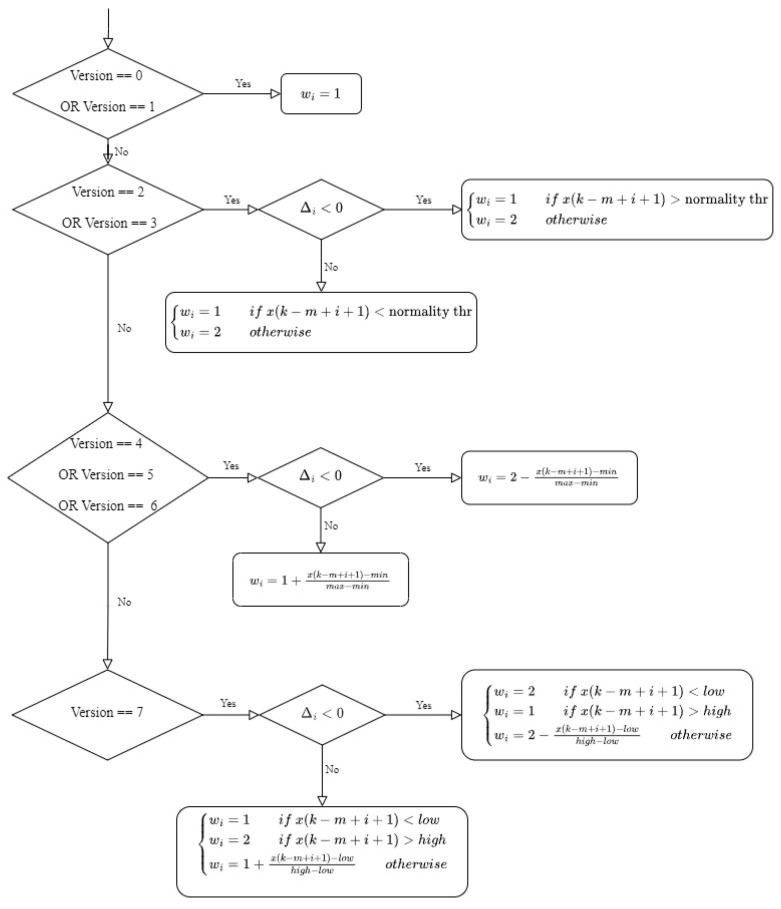
Schematic diagram of the rules that determine the weighting factor *w_i_*.

The rationale for introducing the weighting factor *w_i_* in Equation (2) was to assign a higher weight to a local increase (Δ*_i_* > 0) if the biomarker had a clinically ‘bad’ value and to assign a lower weight to a local increase if the biomarker had a clinically ‘good’ value. Conversely, if the trend exhibited a local decrease (Δ*_i_* < 0), the weight would be higher for ‘good’ values and lower for ‘bad’ values. Accordingly, *w_i_* was introduced in versions #2–7 to capture information about the clinical implications of an increasing/decreasing trend of a biomarker. The value of *w_i_* was determined by comparing the value of *x*(*k − m* + *i* + 1) to clinically defined cut-off thresholds derived from clinical guidelines [15,16,17] (see Table 2). Specifically, Table 2 reports the normality threshold defined above, the min and max values (i.e., the lowest and the highest possible observable clinical values), and the low and high thresholds, (i.e., cut-off values that determine a clinically relevant worsening (e.g., sBP > 160 mmHg, HDL < 1 mmol/L)). A summary of the algorithm for setting the weighting factor *w_i_* is reported in Figure 2. For example, in versions #2 and #3 of the algorithm, *w_i_* was equal to 1 if Δ*_i_* < 0 and *x*(*k* − *m* + *i* + 1) was above the normality threshold (local decrease, but values remaining above the desired range) or if Δ*_i_* > 0 and *x*(*k* − *m* + *i* + 1) was below the normality threshold (local increase, but values remaining below the desired range). In versions #4–7, *w_i_* was defined on a continuous linear scale in the range from 1 to 2 between the *min* and *max* values (versions #4–6) or between the low and high threshold values (version #7) using a similar logic to versions #2 and #3, as reported in detail in Figure 2.

The transition probability matrix *Z* was built using critical levels *m^+^ = m^−^ = m* and a transition probability *r^−^* = 0.5 (i.e., equal probability of a+ or a− in *k +* 1 when *n^−^* < *m^−^* and *n^+^* < *m^+^*). The matrix *Z_inf_* in (1) was estimated as *Z^l^*, with *l* = 10,000. The vector *q_c_* was estimated as the frequency distribution of all the 2*^m^* possible sequences of states in a window of length *m*, as previously introduced. The output variable of the inclination (*I*) was computed as the ratio *P_c_^−^/P^−^* and was defined in the range from 0 to 2. Based on the value of *I*, one of the three outcomes described in Section 2.2.1 can be obtained based on the following rules:

- If *I* > 1 + δ (condition *P_c_^−^ > P^−^*), the predicted final state was collapse;
- If *I* < 1 − δ (condition *P_c_^−^ < P^−^*), the predicted final state was survival;
- If 1 − δ ≤ *I* ≤ 1 + δ (condition *P_c_^−^~P^−^*), no clear inclination was defined. In this case, to predict the final state, the percentage of − and + states in the first and second half of the series was computed, and the predicted final state of the system was set as collapse if the percentage of − in the second half of the series was higher than that in the first one, and it was set as survival otherwise.

In this first exploratory study, the parameter δ was empirically set equal to 0.1 to account for the inherent uncertainty of the clinical data, whereas in the original formulation, δ was set equal to 0 [12,13]. The algorithm was implemented in MATLAB R2020a.

**Figure 3:**
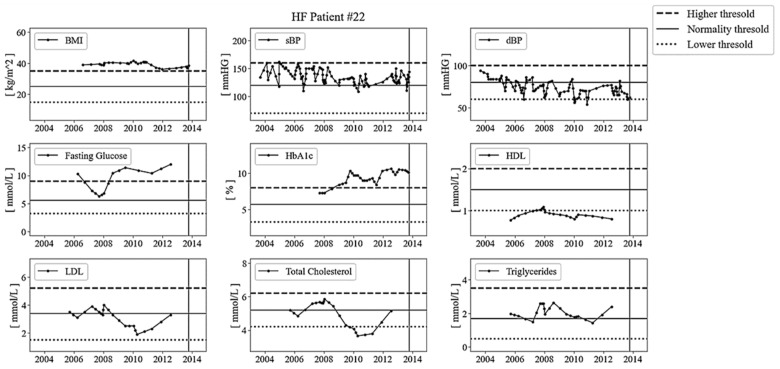
Example of biomarkers time series from one of the patients with HF. The solid vertical line indicates the date of onset (HF diagnosis: 8 October 2013).

The eight versions of the algorithm defined in Table 1 were compared using a dataset from 40 patients (20 with HF, 20 with no HF randomly selected from the 698 patients in our dataset), in which the final state of each of the biomarker time series was manually labeled by two expert physicians (K.K, A.K) as collapse or survival based on clinical considerations. An example is shown in Figure 3. The figure shows all the longitudinal measures of biomarkers available from 2004 to 2014 for a patient diagnosed with HF in 2013. In this example, BMI, sBP, fasting glucose, triglycerides, HDL, and HbA1c were labeled as collapse (i.e., their values were outside the desired range or show a trend towards clinical worsening), whereas dBP, LDL, and total cholesterol were labeled as survival (i.e., their values show a trend towards clinical improvement). For each of the nine biomarkers, Figure 3 shows the time series (including original values and interpolated values), the normality threshold (solid horizontal line), the high threshold (dashed horizontal line), and the low threshold (dotted horizontal line). To compare the eight versions of the algorithm summarized in Table 1, each of the algorithms was used to classify each biomarker (as measured in a time window up to one year before HF onset) as predicted survival/collapse. The accuracy, sensitivity, specificity, and positive predictive value (PPV) of the eight versions of the algorithm were computed for each biomarker across the 40 realizations and then averaged across biomarkers.

### 2.3. Classification Using Machine Learning

A machine learning classifier was trained on the final states of biomarkers predicted using the version of inclination analysis with the highest accuracy, as measured using the methodology described in Section 2.2.2. Specifically, for the 698 patients in our dataset, the predicted final states of the nine biomarkers were used as input features, whereas the future onset of disease (HF/no HF) was used as the output class. As a benchmark, a conventional classifier trained using the average values of biomarkers as input features was considered and compared to one trained using the predicted final states of biomarkers.

The dataset was randomly split into a training (75%) and test set (25%) using stratification. Preliminary analysis of several machine learning algorithms (i.e., decision tree (DT), support vector machines, random forest, and logistic regression) revealed no substantial differences in classification performance. Therefore, in this first study, a decision tree (DT) algorithm was used to assess classification performance and get insight into the rules that determined the prediction of HF onset. The maximum depth of the tree was set at 3 as a trade-off between accuracy and overfitting following preliminary analysis. Hold-out cross validation of the model was performed using 40 randomly shuffled versions of the training and test set. The performance of the DT classifier was addressed by computing the average values of accuracy, recall, specificity, PPV, negative predictive value (NPV), and the area under the receiver operating curve (AUC) across the 40 realizations of training and test sets. The DT algorithm was implemented in Python 3.7 using the scikit-learn library.

## 3. Results

### 3.1. Characterization of the Study Sample

The observed distributions of biomarker measures in patients who will develop HF and who will not develop HF are summarized in Table 3. On average, patients in both groups tend to have mean values (averaged across 5 or more measurements in time) of BMI, sBP, fasting glucose, HbA1c, and HDL outside the clinically desired range, whereas in both groups the mean values of dBP, LDL, total cholesterol, and triglycerides tend to be within the desired range, as defined by the normality thresholds in Table 2. Patients who will develop HF tend to exhibit worse mean values of BMI, sBP, fasting glucose, HbA1c, HDL, and triglycerides and better mean values of dBP, LDL, and total cholesterol compared to patients who will not develop HF. Statistical analysis showed that all the observed differences in mean biomarkers between patients in the HF and no HF group were statistically significant (*t*-test, *p* << 0.01), except for triglycerides (*t*-test, *p* = 0.083). In terms of BMI, patients in the HF group have a higher prevalence of obesity (i.e., BMI ≥ 30 kg/m^2^) and overweight conditions (i.e., 25 kg/m^2^ ≤ BMI < 30 kg/m^2^) than patients in the no HF group. Specifically, about 90% of patients in the HF group are overweight compared to a total of about 80% in the no HF group, with a higher percentage of obese patients in the HF group compared to the no HF group (i.e., 58.2% vs. 44.1%). The higher values of sBP, fasting glucose, and HbA1c in patients with HF may be associated with the fact that a higher percentage of patients with HF in our sample have hypertension and diabetes mellitus compared to those with no HF (i.e., 65.6% vs. 58.2% have hypertension and 87.4% vs. 69.9% have diabetes mellitus).

### 3.2. Characterization of Inclination Analysis

Table 4 shows the performances of the eight versions of the inclination analysis algorithm tested here. The highest performance metrics are obtained using version #7 (i.e., the one in which the weighting factor *w* is scaled between the lowest and highest acceptable clinical values (low and high thresholds)), with sensitivity and specificity around 0.90 and PPV of about 0.83. A slightly lower performance was observed for version #5, in which *w_i_* is scaled using minimum and maximum possible observed clinical values (min and max values). Both versions #5 and #7 consider information about an increase/decrease (but not about the magnitude of the increase) and information about the distance of the observed biomarker values from clinically relevant limits reported in Table 2. Intermediate values of the performance are observed for versions #2 and #3, in which fixed values of *w* are used to account for biomarker values within or outside the clinically desirable range. The lowest performance metrics are observed with the original algorithm (version #0), with a sensitivity slightly above 0.6, specificity below 0.5, and PPV of about 0.53. The low observed performance of version #0 is likely due to the fact that the original algorithm uses only information about a biomarker increase/decrease without addressing the measured values in relation to the clinically meaningful cut-off values shown in Table 2. For the same reason, a low performance is observed using version #1 that adds information about the time variable by computing the area under the time series, but it does not use a weighting factor, *w_i_*, to assess the biomarker values in relation to clinically meaningful values. In version #6, a low performance is observed, mainly due to the use of a longer window (*m* = 4) and a related decrease in reliability of estimates compared to *m* = 3 due to a lower number of window observations (i.e., 6 vs. 7 sliding windows on a time series of 9 values, respectively) for a higher number of possible states (i.e., 14 vs. 7 unstable states if *m^−^* = *m^+^* = *m*, respectively). Therefore, the optimal settings are those in version #7 (*m* = 3; Δ = +/−1; W defined in the range 1–2 using the low/high thr values) and will be used hereafter.

Table 5 reports the percentage of patients in each group with a predicted collapse for each biomarker. Patients with HF tend to have a higher percentage of predicted collapse for BMI, sBP, fasting glucose, HbA1c, and HDL and a lower percentage of predicted collapse for dBP, LDL, and total cholesterol compared to patients with no HF. A statistical analysis (Chi-square test) showed that the observed differences in the percentage of predicted collapse for BMI, sBP, fasting glucose, HbA1c, HDL, LDL, and total cholesterol between HF and no HF are significant (*p* < 0.05), whereas the ones observed for dBP and triglycerides were not significant (*p* = 0.08 and *p* = 1.00, respectively).

The results shown in Table 5 are analyzed in more detail by introducing a ‘similarity score’, which is defined as the number of biomarkers that mimic the average behaviour shown in Table 5. Therefore, considering that only 7 out of 9 biomarkers show statistically significant differences between the two groups, the similarity score is defined on a range from 0 to 7. For example, for a given patient in the HF group, the similarity score will be equal to 7 if the inclination analysis algorithm predicts a collapse for BMI, sBP, fasting glucose, HbA1c, and HDL and a survival for LDL and total cholesterol, whereas the similarity score will be equal to 0 in case of opposite predictions (collapse for LDL and total cholesterol and survival for all the other biomarkers). In the HF group, 252 out of 349 patients (72.2%) have a similarity score equal to 6 or 7, and 316 out of 349 patients (90.5%) have a similarity score equal to or higher than 5. In the no HF group, 177 out of 349 patients (50.7%) have a similarity score equal to or higher than 6, and 251 (71.9%) patients have a similarity score equal to or higher than 5, indicating that patients in the HF group are more aligned with the average behaviour shown in Table 5 (i.e., a tendency towards a collapse for BMI, sBP, fasting glucose, HbA1c, and HDL and a tendency towards survival for LDL and total cholesterol) compared to those in the no HF group.

### 3.3. Classification Results

**Figure 4:**
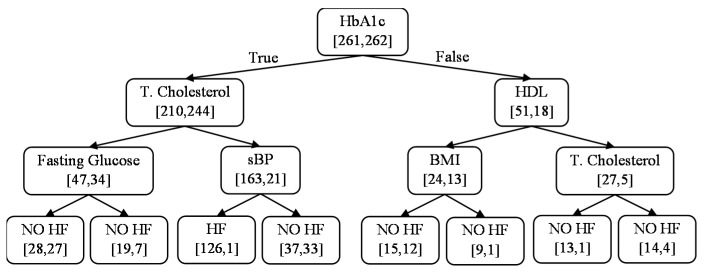
Example of DT trained on inclination analysis data from one of the 40 iterations (true = predicted ‘collapse’). Performance: accuracy = 0.63, recall = 0.79, specificity = 0.47, PPV = 0.60, NPV = 0.70, and AUC = 0.63.

An example of DT obtained from one of the 40 iterations is shown in Figure 4. This model predicts the HF class in patients with a predicted collapse (true) for HbA1c and sBP and predicted survival (false) for total cholesterol, in line with the results shown in Table 5. This finding is consistently observed across the 40 iterations. Specifically, the more frequently occurring features in the trained models are, on average, HbA1c, total cholesterol, sBP, and HDL.

Table 6 shows the average performance measures of DT classifiers observed across the 40 iterations used as input features for either the predicted final states or the average values of the biomarkers computed across 5 or more measurements in time. On average, the DTs trained on the predicted final states have a similar accuracy, PPV, and AUC to those trained on the average values of biomarkers (*t*-test: *p* = 0.05, 0.07, and 0.13 for accuracy, PPV, and AUC, respectively). Noticeably, a significant improvement in recall (at the expenses of specificity) and NPV is observed for classifiers trained using the predicted final states (*t*-test: *p*~10^−12^ and ~10^−4^ for recall and NPV, respectively) compared to those trained on the average values.

## 4. Discussion

This exploratory study introduces an original approach based on inclination analysis to predict the onset of HF from longitudinal measurements of clinical biomarkers extracted from primary care EMRs.

An analysis of several versions of the inclination analysis algorithm indicates that the final state of biomarkers is predicted more accurately by algorithms that use a moving window of length (*m* = 3) and that estimate the local state of each biomarker based on a measure of distance (i.e., the weighting factor *w*) defined between the lowest and highest acceptable clinical values (low and high thresholds), as reported in Table 2. Specifically, the best performing algorithm (version #7) shows sensitivity equal to 0.89, specificity equal to 0.90, and PPV equal to 0.83, that is a substantially higher performance compared to the original version (version #0: sensitivity = 0.62, specificity = 0.45, PPV = 0.53). The analysis of the percentage of predicted collapse in patients with HF and with no HF using version #7 of the algorithm further validates this approach. Specifically, patients with HF tend to have a higher percentage of collapse predictions (Table 5) for the same biomarkers, in which worse average values are observed (i.e., BMI, sBP, fasting glucose, HbA1c, triglycerides, and HDL, as shown in Table 3).

It should be noted that a predicted collapse for a given biomarker indicates a trend over time for that biomarker to become steadily worse (i.e., inclination analysis provides an additional piece of information compared to the analysis of the average values in Table 3). Specifically, in the majority of patients with HF, most of the biomarkers tend to generate collapse predictions (e.g., more than 90% of patients in the HF group have a similarity score ≥ 5 out of 7 compared to about 72% of patients in the no HF group). The relatively high percentage of patients in the no HF group showing tendency towards collapse may be related to the fact that, in the dataset used here, patients with no HF may develop other diseases and tend to exhibit some abnormalities in the observed biomarkers (e.g., BMI, sBP, HDL, fasting glucose, and HbA1c, see Table 3). An inclination analysis confirms a higher tendency towards collapse for BMI, sBP, fasting glucose, HbA1c, and HDL and a higher tendency towards survival for LDL and total cholesterol in both groups, but different behaviour is observed between the HF and no HF groups (Table 5). For example, the analysis of BMI indicates a higher tendency towards overweightness and obesity in the HF group compared to the no HF group, in line with evidence from the literature [18]. Predictions from the inclination analysis further confirm this finding as the BMI measurements tend towards collapse in a higher percentage of patients in the HF group compared to the no HF group (i.e., 79% vs. 71%). According to 2018 data, 63.1% of Canadians 18 and older were classified as overweight (36.3%) or obese (26.8%) [19]. In our dataset, higher percentages are observed, mainly due to the fact that our sample is derived from a database of primary care EMRs, including records from patients who, on average, have more diseases/conditions compared the general adult population. Similarly, the prevalence of hypertension and diabetes in our dataset is higher than in the adult Canadian population. Hypertension (defined as sBP ≥ 140 mmHg or dBP ≥ 90 mmHg as in the CPCSSN disease definition used here) is reported in one in four adults (i.e., about 24%) [20,21], whereas diabetes affects about 10% of adults in Canada, with an increasing prevalence with increasing age [22]. In our dataset, the percentage of patients with HF diagnosed with hypertension or diabetes before the onset of HF is about 66% and 87%, respectively, compared to about 58% and 70%, respectively, in the no HF group. The fact that HF patients tend to exhibit a higher prevalence of hypertension and diabetes is in line with the fact that these conditions are known risk factors for HF [23,24]. In line with these percentages, patients with HF exhibit increased sBP, fasting glucose, and HbA1c and an increased tendency towards collapse for these biomarkers compared to patients with no HF, in line with evidence from the literature [24,25].

Regarding the behaviour of LDL and total cholesterol in patients with HF, these biomarkers show apparently counterintuitive trends as they tend to be lower (i.e., better) in the HF group compared to the no HF group. Specifically, a collapse is predicted in a minor proportion of patients with HF (i.e., about 12% (Table 5)). This trend is also reflected by a significantly lower percentage of predicted collapse for LDL and total cholesterol. This might be due to the fact that patients with HF are characterized by having hyperlipidemia as a possible precursor of the disease, and as such, they are likely to be on cholesterol-lowering drugs, such as statins (i.e., LDL-lowering medicines with known protective effects on the heart), years before the diagnosis of HF [26]. It is important to note that, frequently, a diagnosis of HF occurs late with the disease at an already advanced stage and a mid-range or reduced ejection fraction [27]. As such, for some patients, the date of onset extracted from the EMR might be an inexact estimate of the actual onset of the disease, and the signs and symptoms of disease might have been actually developed years before, as suggested by the analysis of biomarkers related to the metabolism of lipids, such as LDL and total cholesterol.

The DT classifier trained on the predicted final states of biomarkers has similar accuracy as the one trained on the average values of biomarkers (0.58 vs. 0.57). Overall, relatively low levels of classification accuracy are observed in this study (on average, 0.58) due to a combination of factors. One of the main reasons is related to the use of routinely collected clinical biomarkers as opposed to disease-specific biomarkers commonly used in previous HF prediction investigations (e.g., [5,6,7,8,9,10,11]). The nine biomarkers used here are not specific to HF or heart disease but are more generally related to common conditions (e.g., elevated blood pressure, hyperlipidemia, and hyperglycemia). Therefore, they may have relatively low predictive performance in relation to HF, especially in light of the difficult diagnosis and clinical definition of this disease. However, the use of widely available biomarkers for model training leads to models that can be applied to a large population (i.e., to primary care patients who undergo routine lab testing and examinations). For this reason, the approach shown here is also potentially applicable to model other chronic diseases, such as diabetes, hypertension, chronic kidney disease, or chronic respiratory diseases. The classification accuracy observed here is in line with the findings from other studies that used DT algorithms. For example, a previous study reported that the sensitivity = 0.46, specificity = 0.82, and PPV = 0.62 for DT trained on a dataset including a combination of 34 general and disease-specific features [6]. Similarly, in [7], the estimated AUC of DT ranged from 0.65 to 0.75 using different kinds of data, including a combination of administrative claims and data from EMRs.

Noticeably, a substantially increased recall (0.69 vs. 0.53) and a significantly improved NPV (0.60 vs. 0.55) are observed (Table 6) when the DT is trained on the predicted final states of biomarkers. It is acknowledged that the observed improvement in recall brings along a decrease in specificity as the accuracies of the two DTs are similar. However, a higher recall is potentially important for the sake of HF prediction as it lowers the false negative rate and, therefore, the Type II error, which is more critical than a Type I error from a disease prediction and prevention point of view. Therefore, patients who will develop HF are more likely to be identified as patients at risk of developing HF (and less likely to be misclassified as not being at risk) if a DT model is trained on the inclination analysis predictions compared to a model trained on the average values of biomarkers. Moreover, the DT trained on the predicted final states has higher NPV than the DT trained on averaged biomarkers, indicating that a lower proportion of patients who are identified as not being at risk will actually develop HF if a classifier trained on the output of inclination analysis is used, therefore reducing the proportion of at-risk patients who would not be identified and would not be offered preventive interventions.

In addition, the use of an inclination analysis could potentially open opportunities for a real-time prediction of risk on an individualized basis. Specifically, a routine collection of clinical biomarkers as the one considered here in primary care settings could help develop predictions of biomarkers towards survival/collapse, as well as predictions of patient health status towards a risk of HF that can be updated at each clinical encounter. This is particularly relevant considering the progressive and degenerative nature of HF and the consequently late diagnosis. Future research will be necessary to address the performance of the algorithm as a function of the length of the time series and to investigate the ability of the algorithm to monitor the individual risk and possibly support an earlier identification of HF. It will also be important to investigate the performance of the approach on different high-prevalence chronic diseases, such as type 2 diabetes or respiratory chronic disease. This study has some limitations. First, the requirement of five or more readings for each of the nine biomarkers has substantially limited the study sample. It will be important to validate the approach on a larger dataset, including a higher number of measurements of time to study the performance of the algorithm as a function of the number of measurements and evaluate the algorithm’s ability to support an early detection of the risk. Towards a future implementation of real-time risk prediction using the proposed approach, it will be important to investigate the inclination analysis algorithm in more detail and define requirements related to the optimal number of samples and the optimal sampling strategy. For example, it will be useful to investigate the performance of the algorithm in a larger sample of manually labeled records to include a higher number of records with a final ‘survival’ state (for example, for some biomarkers, less than 10–20% of records may be associated with a final ‘survival’ state, as suggested by Table 5). It will also be important to analyze the optimal sampling as a function of the inherent dynamics of biomarkers (e.g., shorter intervals may be necessary for laboratory biomarkers, whereas longer intervals may be sufficient for BMI).

In addition, the analysis of DT performance is based only on one out of the eight versions of the algorithm tested here, but for a more specific characterization of inclination analysis, it would be interesting to understand the influence of other versions of the algorithm with a similar accuracy (e.g., version #5), as well as to develop novel versions of the inclination analysis algorithm with a potentially higher performance. Moreover, it would be important to assess the performance of classifiers obtained using other machine learning algorithms or a subset of input features (for example, by using natively explainable methods or post hoc explainability techniques, such as partial dependence plots or Shapley additive explanations [28,29,30,31]). Last, but not least, it will be important to assess how the predictions generated by an inclination analysis could help generate personalized recommendations for individuals with a higher risk, as identified by a real-time analysis of EMR data. For example, the analysis of counterfactual explanations (i.e., minimal changes in biomarkers required to lower the individual risk [32,33,34,35]) could help identify individualized recommendations that may help slow down or reverse the inclination of specific biomarkers and, therefore, reduce the risk of developing the disease, supporting a personalized prevention of the disease.

## 5. Conclusions

The results of this exploratory study, although preliminary, are encouraging and provide the basis for further investigations towards more a specific validation of the proposed approach. Future studies will be necessary to further investigate the use of an inclination analysis for HF prediction and, more generally, for chronic disease prediction. It will be important to address the role of each biomarker and the related predicted final states in determining the risk of HF. It will also be interesting to compare the performance of the proposed approach with other algorithms able to deal with longitudinal data, such as dynamical models or regression algorithms. In addition, new insights could be obtained by combining data from EMRs and data from patient monitoring devices to fully understand the ability of the binary predictions from an inclination analysis to characterize the trajectories of biomarkers in a clinically meaningful way and support the patient towards the achievement of individual target biomarker values for the sake of risk reduction.

## Figures and Tables

**Table 1:** Settings used in the eight tested versions of the inclination analysis algorithm, including the original version (#0) and seven new versions (versions #1–7).

#	m	Δ*_i_* Definition	W_i_ Definition
0	3	difference	1
1	3	integral-ratio	1
2	3	difference	1 or 2 (norm thr)
3	3	slope	1 or 2 (norm thr)
4	3	slope	Range 1–2 (min/max)
5	3	+/−1	Range 1–2 (min/max)
6	4	+/−1	Range 1–2 (min/max)
7	3	+/−1	Range 1–2 (low/high thr)

**Table 2:** Clinically defined cut-off values for the nine biomarkers.

Biomarker	Unit of Measure	Low Thr	Normality Thr	High Thr	Min	Max
BMI	Kg/m^2^	15	25	35	10	60
dBP	mmHg	60	80	100	20	192
sBP	mmHg	70	120	160	50	266
Fasting Glucose	mmol/L	3.2	5.6	9	1.3	23
HbA1c	%	3.3	5.7	8	0.05	18.5
HDL	mmol/L	1	1.5	2	0.6	3
LDL	mmol/L	1.5	3.4	5.2	0.7	8
Total cholesterol	mmol/L	4.2	5.2	6.2	2	13
Triglycerides	mmol/L	0.5	1.7	3.5	0.1	20

**Table 3:** Statistical characterization of biomarkers in patients with HF and with no HF.

		HF				No HF			
Biomarker	Unit of Measure	Mean	SD	Min	Max	Mean	SD	Min	Max
BMI *	Kg/m^2^	32.5	6.64	16.8	57.9	30.2	6.54	17.2	56.5
dBP *	mmHg	73.3	6.89	56.9	92.5	75.7	7.35	57.6	105
sBP *	mmHg	132.8	11.35	102.9	166.7	129.5	10.68	91.0	161.8
Fasting Glucose *	mmol/L	7.6	1.82	4.5	19.5	6.7	1.47	4.0	13.0
HbA1c *	%	7.1	1.16	5.3	12.7	6.6	0.99	3.8	11.6
HDL *	mmol/L	1.2	0.32	0.7	2.3	1.3	0.34	0.7	2.7
LDL *	mmol/L	2.2	0.68	0.9	5.0	2.5	0.75	0.9	4.4
Total cholesterol *	mmol/L	4.2	0.84	2.5	8.0	4.5	0.91	2.6	7.2
Triglycerides	mmol/L	1.7	0.78	0.5	6.4	1.6	0.74	0.5	4.4

* *p* < 0.01.

**Table 4:** Performance of the eight versions of the inclination analysis algorithm.

	0	1	2	3	4	5	6	7
Accuracy	0.56	0.56	0.73	0.71	0.63	0.85	0.58	0.89
Sensitivity	0.62	0.61	0.70	0.66	0.57	0.85	0.56	0.89
Specificity	0.45	0.53	0.76	0.72	0.64	0.89	0.60	0.90
PPV	0.53	0.56	0.65	0.64	0.60	0.78	0.57	0.83

**Table 5:** Percentage of predicted ‘collapse’ for each biomarker in patients with HF and with no HF.

	HF	No HF
BMI *	0.79	0.71
dBP	0.37	0.44
sBP *	0.86	0.78
Fasting Glucose *	0.82	0.69
HbA1c *	0.94	0.82
HDL *	0.81	0.69
LDL *	0.12	0.20
Total cholesterol *	0.12	0.23
Triglycerides	0.37	0.37

* *p* < 0.01.

**Table 6:** Performances of DT classifiers trained on the predicted states and on the average values of biomarkers (*N* = 40 iterations).

	Predicted States Mean (s.d.)	Average Values Mean (s.d.)
Accuracy	0.58 (0.028)	0.57 (0.036)
Recall *	0.69 (0.058)	0.53 (0.11)
Specificity *	0.47 (0.068)	0.61 (0.115)
PPV	0.57 (0.038)	0.59 (0.055)
NPV *	0.60 (0.046)	0.55 (0.054)
AUC	0.58 (0.028)	0.57 (0.034)

* *p* < 0.01.

## Data Availability

This study involves third-party data that cannot be shared because of data sharing agreement restrictions by the data holder. The entire source database is available from the Canadian Primary Care Sentinel Surveillance Network (https://cpcssn.ca/join-cpcssn/for-researchers/, accessed on 23 March 2023) for researchers who meet the criteria for access to deidentified patient data. The minimal dataset used to reach the conclusions drawn in the manuscript can be accessed upon a specific agreement to be signed with the institution holding data access rights (Toronto Metropolitan University).
